# Point-of-Care Tests for Rapid Detection of Porcine Epidemic Diarrhea Virus: A Systematic Review and Meta-Analysis

**DOI:** 10.3390/v14071355

**Published:** 2022-06-21

**Authors:** Renfeng Li, Xiangqin Tian, Junzeng Pang, Linyue Li, Jiakang Yuan, Zhuangzhuang Tian, Ziliang Wang

**Affiliations:** 1 College of Animal Science and Veterinary Medicine, Henan Institute of Science and Technology, Xinxiang 453003, China; lirenfeng@sina.com (R.L.); pjz29716@163.com (J.P.); m18236356245@163.com (L.L.); yuanjiakang0905@163.com (J.Y.); zlw19983511@126.com (Z.W.); 2 Henan Key Laboratory of Medical Tissue Regeneration, Xinxiang Medical University, Xinxiang 453003, China; 3 School of International Education, Xinxiang Medical University, Xinxiang 453003, China; tzz17634295329@163.com

**Keywords:** porcine epidemic diarrhea virus, point-of-care tests, lateral flow immunochromatography assay, nucleic acid isothermal amplification, systematic review, meta-analysis

## Abstract

The timely and accurate diagnosis of porcine epidemic diarrhea virus (PEDV) infection is crucial to reduce the risk of viral transmission. Therefore, the objective of this review was to evaluate the overall diagnostic accuracy of rapid point-of-care tests (POCTs) for PEDV. Studies published before 7 January 2022 were identified by searching PubMed, EMBASE, Springer Link, and Web of Science databases, using subject headings or keywords related to point of care and rapid test diagnostic for PEDV and PED. Two investigators independently extracted data, rated risk of bias, and assessed the quality using the Quality Assessment of Diagnostic Accuracy Studies-2 tool. The bivariate model and the hierarchical summary receiver operating characteristic (HSROC) model were used for performing the meta-analysis. Threshold effect, subgroup analysis, and meta-regression were applied to explore heterogeneity. Of the 2908 records identified, 24 eligible studies involving 3264 specimens were enrolled in the meta-analysis, including 11 studies on evaluation of lateral flow immunochromatography assay (ICA)-based, and 13 on nucleic acid isothermal amplification (NAIA)-based POCTs. The overall pooled sensitivity, specificity and diagnostic odds ratio (DOR) were 0.95 (95% CI: 0.92–0.97), 0.96 (95% CI 0.88–0.99) and 480 (95% CI 111–2074), respectively; for ICA-based POCTs and the corresponding values for NAIA-based, POCTs were 0.97 (95% CI 0.94–0.99), 0.98 (95% CI 0.91–0.99) and 1517 (95% CI 290–7943), respectively. The two tests showed highly comparable and satisfactory diagnostic performance in clinical utility. These results support current recommendations for the use of rapid POC tests when PEDV is suspected.

## 1. Introduction

Porcine epidemic diarrhea virus (PEDV), a member of the *Alphacoronavirus* genus of the *Coronaviridae* family, can induce swine acute gastrointestinal symptoms characterized by dehydration, vomiting, diarrhea, and high mortality in newborn and suckling piglets, resulting in considerable economic damage to the global swine industry [1]. Notably, the evolutionary origin of PEDV from bats and the ability of cross-species transmission pose potential threats to human health [2,3,4]. In fact, the SARS-CoV genome shows evidence of recombination with PEDV at some point in its evolutionary history [5]. In 2016, we isolated a highly virulent PEDV CH/HNQX-3/14 strain from a farm in Henan, China, and identified it as a novel recombination PEDV strain derived from the attenuated vaccine strains (CV777 and DR13) and circulating wild-type strain (CH/ZMDZY/11) [6]. Increasing genetic diversity of PEDV and PEDV coinfection and recombination with other enteric viruses highlight the importance of rapid and accurate diagnosis of PEDV at the early stage for proper disease and outbreak management [7,8,9,10], allowing immediate and precise surveillance, prevention and control measures. Laboratory testing such as enzyme-linked immunosorbent assay (ELISA), polymerase chain reaction (PCR), and immunohistochemistry (IHC) are complex, time-consuming, and require a large quantity of reagents and well-equipped laboratories, which limit their application in low-resource settings.

POCTs are a kind of rapid diagnostic test that can be performed by clinical staff without laboratory training at the site of sample collection. To date, the rapid POCTs for the diagnosis of PEDV based on lateral flow immunochromatography assay (ICA) and nucleic acid isothermal amplification (NAIA) technologies have been developed and these tests have the potential to allow earlier diagnosis and reduce the spread of PEDV, particularly in many settings where laboratory-based tests are usually not available, filling the gaps in diagnosis of PEDV. However, it is not clear whether the scientific evidence supports the continued use of the POCTs for PEDV. Prospective and comparative evaluations of rapid POCTs for PEDV in clinically relevant settings are urgently needed. The primary objective of this systematic review and meta-analysis is therefore to evaluate the diagnostic accuracy of ICA- and NAIA-based POCTs for the diagnosis of PEDV infection.

## 2. Materials and Methods

### 2.1. Search Strategy

The present systematic review and meta-analysis were conducted in accordance with the preferred reporting items for systematic reviews and meta-analysis (PRISMA) guideline for systematic reviews of diagnostic tests by the Cochrane Collaboration [11]. We systematically searched PubMed, EMBASE, SpringerLink, and Web of Science databases using the following combinations of search terms: (“porcine epidemic diarrhea” OR “porcine epidemic diarrhea virus” OR “PEDV”) AND (“point-of-care” OR “rapid test” OR “diagnosis” OR “diagnostic” OR “detection”). No restrictions were applied concerning language. Since this study was a systematic review of published articles, neither informed consent nor ethics approval was required. We also manually searched the relevant references to identify potential articles. The most recent search was performed on 7 January 2022.

### 2.2. Study Selection and Data Extraction

The full text of the studies deemed relevant were reviewed to determine eligibility. The studies were included using the following criteria: (1) peer-reviewed original articles on POCTs for PEDV; (2) full text is available; (3) provides enough information to determine the number of true-positive, false-positive, false-negative, and true-negative on POCTs for PEDV (performed on clinical samples) relative to a reference test; (4) test accuracy studies of any design that evaluated antigen or molecular tests for PEDV suitable for a point-of-care setting. We excluded review articles, editorials, case reports, modeling studies, expert opinions, animal experiments, preprints, and studies that did not perform POCTs or rapid diagnostic tests for PEDV. Reports that presented duplicate data and studies with insufficient data to construct 2 × 2 contingency tables were also excluded. Two reviewers (L.L. and J.P.) independently screened the literature search and assessed each study for inclusion using the Quality Assessment of Diagnostic Accuracy Studies 2 tool (QUADAS-2) recommended by the Cochrane Collaboration [12]. Any disagreements on study inclusion through discussion with a third review author (R.L.).

### 2.3. Data Analysis

If available, the following data were collected from each study: numbers of true-positive, false-negative, true-negative and false-positive results of each individual POCT for PEDV testing versus the reference standard used for comparison. If the study did not directly provide these raw numbers, we used reported sensitivity, specificity, PPV, NPV, and total sample size to back-calculate to obtain integer numbers or contacted the corresponding author in an effort to retrieve the missing information.

We used the bivariate random-effects model and the hierarchical summary receiver operating characteristic model for performing the meta-analysis as well as the pooled sensitivity and specificity, positive likelihood ratio (PLR), negative likelihood ratio (NLR), and diagnostic odds ratio (DOR). We also graphed the summary receiver operating characteristic (SROC) curve to determine the overall diagnostic performance of the index tests. The closer the curve approaches the upper-left corner, the higher the overall performance is [13]. We examined heterogeneity across the studies by visually inspecting the forest plots of sensitivity and specificity and further assessed using Cochran’s Q statistic and the *I*^2^ test; *p* value < 0.05 (Q statistic) and/or *I*^2^ > 50% was considered statistically significant heterogeneity [14]. If heterogeneity existed, subgroup analysis and meta-regression analysis were performed to explore the potential sources of heterogeneity. Publication bias was evaluated using linear regression tests of Deeks’ funnel plot asymmetry; a *p* value of less than 0.1 for the slope coefficient was considered to indicate significant asymmetry and thus publication bias [15]. The pooled Cohen’s kappa value was calculated for evaluating the agreement between different tests. Furthermore, the clinical utility was assessed using Fagan plots [16] and likelihood ratio scattergrams [17]. All analyses were performed by using Review Manager version 5.4 (Copenhagen: Nordic Cochrane Centre, The Cochrane Collaboration, Denmark, 2020), STATA version 17.0 (Stata Corporation, College Station, TX, USA) with the MIDAS module, and Meta-DISc version 1.4. *p*-values < 0.05 were considered statistically significant.

## 3. Results

### 3.1. Literature Search

**Figure 1:**
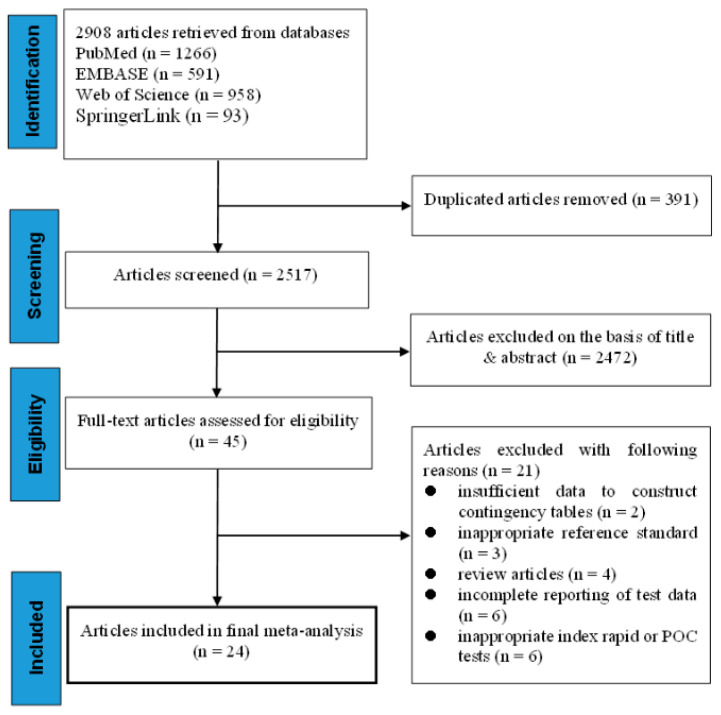
PRISMA flow diagram showing the number of records initially identified and that were subsequently excluded or included in the meta-analysis on the performance and operational characteristics of ICA- and NAIA-based POCTs for PEDV. PRISMA, preferred reporting items for systematic reviews and meta-analysis.

The literature search process is shown in Figure 1. We initially identified 1266 articles from PubMed, 591 articles from EMBASE, 958 articles from Web of Science, and 93 articles from SpringerLink. After removing duplicate articles (391 articles), 2424 publications were kept for screening, of which 2379 articles were subsequently discarded after reviewing titles and abstracts. The remaining 45 articles were eligible for reading the full text, and 21 of them were excluded because of insufficient data to construct 2 × 2 contingency tables (*n* = 2), inappropriate reference standard (*n* = 3), inappropriate index POC tests (*n* = 6), incomplete reporting of test data (*n* = 6) or review articles (*n* = 4). Finally, 24 unique articles addressing at least one of the POCTs or rapid testing for PEDV were included for quantitative data synthesis and meta-analysis (Figure 1).

### 3.2. Characteristics and Quality Assessment

**Figure 2:**
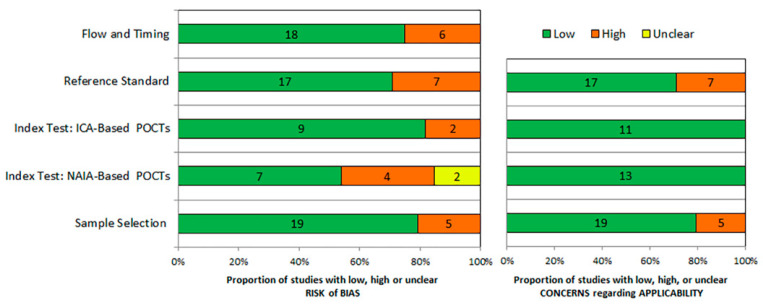
Risk of bias and applicability concerns graph: review of authors’ judgements about each domain presented as percentages across included studies. Numbers in the bars indicate the number of studies.

The data sets were extracted from the 24 articles and consisted of 3264 samples. Among the 24 studies included, 11 studies evaluated the diagnostic performance of ICA-based POCTs and 13 studies evaluated NAIA-based POCTs. All of the studies were published from 2015 to 2021, and the majority (19/24, 79.2%) of these studies were conducted in China, and the remaining studies were from Korea, Japan and Egypt. Table 1 summarized the data extracted from the studies, including first author, year of publication, type of specimen, reference standard, sample size, and numbers of true positive, false positive, false negative, and true negative. We assessed the quality of all the available studies according to QUADAS-2. The results demonstrated that the overall quality of the enrolled studies was acceptable. The risk of bias and applicability concerns graph for the included studies are shown in Figure 2 and Table S1 (Supplementary File).

### 3.3. Diagnostic Performance of POCTs for PEDV

**Figure 3:**
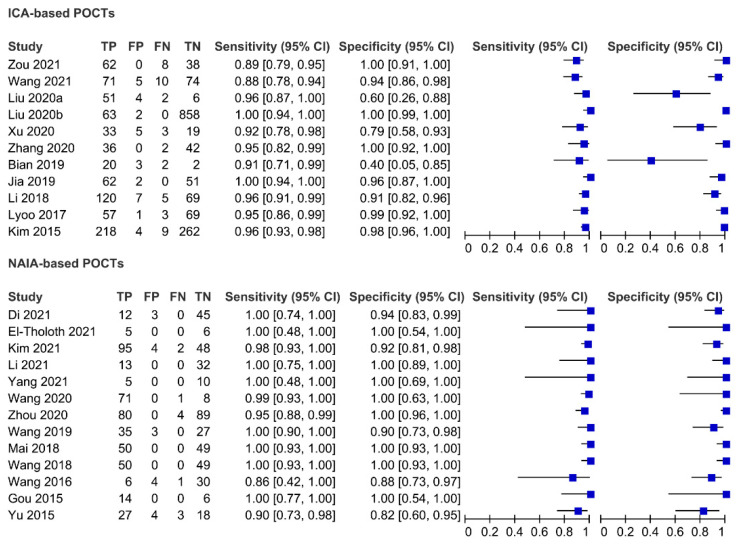
Forest plots of summary sensitivity and specificity of ICA- and NAIA-based POCTs in diagnosis of PEDV. Numbers are pooled estimates with 95% confidence intervals (CIs) in brackets.

**Figure 4:**
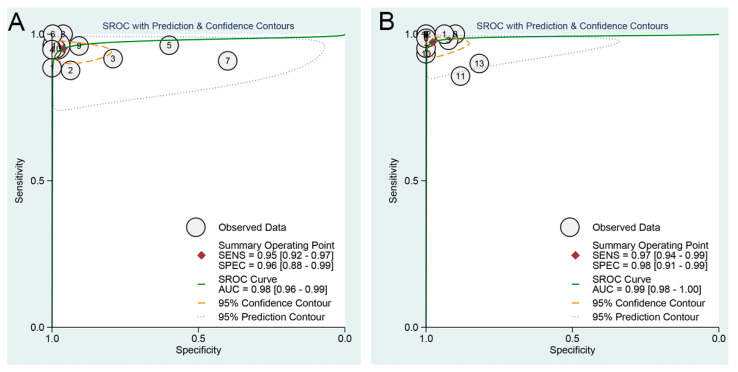
Summary receiver operating characteristic curves showing the pooled sensitivity and specificity of (**A**) ICA- and (**B**) NAIA-based POCTs in diagnosis of PEDV.

**Figure 5:**
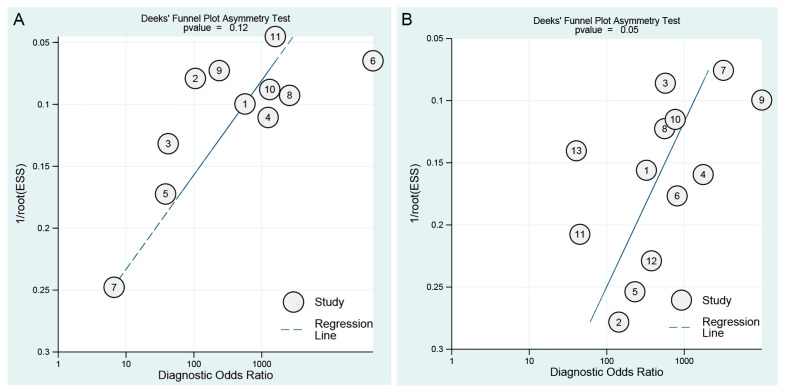
Deeks’ funnel plot asymmetry test for publication bias in different tests for PEDV. (**A**) ICA-based POCTs; (**B**) NAIA-based POCTs. ESS = effective sample size.

Figure 3 showed paired forest plots of the sensitivity and specificity of ICA- and NAIA-based POCTs in the diagnosis of PEDV infection. For ICA-based POCTs, the sensitivities and specificities of the individual studies ranged from 0.88 to 1.00 and 0.40 to 1.00, respectively. The Higgins *I*^2^ statistics demonstrated substantial heterogeneity in terms of both the sensitivity (*I*^2^ = 65.17%, 95% CI 42.80–87.54%, Q = 28.71, *p* = 0.00) and specificity (*I*^2^ = 92.78%, 95% CI 89.75–95.81%, Q = 138.52, *p* = 0.00). ICA-based POCTs had a higher agreement with RT-PCR than real-time RT-PCR for PEDV testing (Cohen’s kappa statistic of 0.94 vs. 0.92, both *p* < 0.001). The overall sensitivity of the studies included in the analysis was estimated from the bivariate random effects model to be 0.95 (95% CI 0.92–0.97). Similarly, the overall specificity was estimated to be 0.96 (95% CI 0.88–0.99). The pooled PLR, NLR and DOR were 24.5 (95% CI 7.4–81.0), 0.05 (95% CI 0.03–0.08), and 480 (95% CI 111–2074), respectively. The area under the summary ROC curve (AUC) was 0.98 (95% CI 0.96–0.99) (Table 2 and Figure 4A), suggesting a high accuracy of ICA-based POCTs used as the diagnosis of PEDV infection. Furthermore, we observed that the proportion of heterogeneity, likely due to the threshold effect, was 0.17 and the Spearman correlation coefficient was −0.209 (*p* = 0.537), indicating that the threshold effect was not the source of heterogeneity. We evaluated publication bias through Deeks’ funnel plot asymmetry test, which clearly demonstrated that no significant publication bias existed in this meta-analysis (*p* = 0.12) (Figure 5A).

For NAIA-based POCT, 13 articles published since 2015 were included in our meta-analysis. The sensitivities and specificities of the individual studies ranged from 0.86 to 1.00 and 0.82 to 1.00 (Figure 3), respectively. The Higgins *I*^2^ statistics demonstrated that there was a moderate heterogeneity in terms of the sensitivity (*I*^2^ = 38.74%, 95% CI 0.00–78.95%, Q = 19.59, *p* = 0.08) and a high heterogeneity for specificity (*I*^2^ = 68.22%, 95% CI 49.89–86.56%, Q = 37.76, *p* = 0.00). The agreement of NAIA-based POCTs with RT-PCR and real-time RT-PCR were 0.93 and 0.92 (both *p* < 0.001), respectively. The pooled sensitivity and specificity were 0.97 (95% CI 0.94–0.99) and 0.98 (95% CI 0.91–0.99), respectively. The PLR and NLR were 42.4 (95% CI 10.9–164.9) and 0.03 (95% CI 0.01–0.06), respectively. The DOR was 1517 (95% CI 290–7943). The AUC was 0.99 (95% CI 0.98–1.00) (Table 2 and Figure 4B), which suggested NAIA-based POCTs have a relatively high accuracy for PEDV testing. Additionally, the proportion of heterogeneity likely due to threshold effect was 0.19 and the Spearman correlation coefficient was −0.369 (*p* = 0.214), indicating that the threshold effect was not the source of heterogeneity. Deeks’ funnel plot asymmetry test was adopted to detect the publication bias. As shown in Figure 5B, a *p* value of 0.05 indicated significant publication bias in the current meta-analysis.

Compared to the ICA-based POCTs, the NAIAA-based POCTs showed better summary diagnostic performance in sensitivity (0.95, 95% CI 0.92–0.97 vs. 0.97, 95% CI 0.94–0.99), specificity (0.96, 95% CI 0.88–0.99 vs. 0.98, 95% CI 0.91–0.99), and DOR (480, 95% CI 111–2074 vs. 1517, 95% CI 290–7943). As can also be observed in the HSROC plots (Supplementary File Figure S1), although both the ICA- and NAIA-based POCTs showed highly satisfactory summary point estimates in the HSROC, the NAIAA-based POCTs illustrated a narrower confidence region and a more restricted prediction region than ICA-based POCTs.

### 3.4. Heterogeneity Exploration

For ICA-based POCT, further subgroup analysis and meta-regression were conducted to find probable sources of heterogeneity. The results showed that none of the covariates, including type of sample (*p* = 0.90), sample size (*p* = 0.08), and reference standard (*p* = 0.14), were significant factors affecting heterogeneity in the meta-regression analysis using the Joint model (Table 3). When comparing sensitivity and specificity estimates with the covariates, we found that the studies with sample size ≥ 100 showed a slightly better sensitivity and a significantly higher specificity compared to that <100 of sample size (sensitivity: 0.96, 95% CI 0.93–0.98 vs. 0.94, 95% CI 0.89–0.99, *p* = 0.05; specificity: 0.98, 95% CI 0.96–1.00 vs. 0.82, 95% CI 0.60–1.00, *p* = 0.02). Interestingly, the studies conducted with feces samples appeared to have a significantly lower pooled sensitivity when compared to that of other types of sample including rectal swab, small intestine, colostrum, and serum (0.95, 95% CI 0.91–0.98 vs. 0.96, 95% CI 0.92–0.99, *p* = 0.00), and the studies conducted with ELISA as reference standard showed a higher pooled sensitivity (0.96, 95% CI 0.92–1.00 vs. 0.94, 95% CI 0.92–0.97, *p* = 0.19) and a lower specificity than that of other reference standards including RT-PCR and real-time RT-PCR (0.81 95% CI 0.46–1.00 vs. 0.97 95% CI 0.94–1.00, *p* = 0.21) (Table 3 and Supplementary File Figure S2A).

Regarding NAAT-based POCTs, subgroup analysis and meta-regression were performed according to the type of sample, sample size, reference standard, and type of assay. For the subgroup based on sample type, the studies conducted on feces sample obtained comparable sensitivity and specificity with those on other types of samples (sensitivity: 0.97, 95% CI 0.94–0.99 vs. 0.98, 95% CI 0.95–1.00, *p* = 0.50; specificity: 0.97, 95% CI 0.93–1.00 vs. 0.98, 95% CI 0.94–1.00, *p* = 0.44). The studies with sample size ≥100 showed slightly lower sensitivity but higher specificity compared to that of <100 (sensitivity: 0.97, 95% CI 0.93–1.00 vs. 0.98, 95% CI 0.95–1.00, *p* = 0.18; specificity: 0.98, 95% CI 0.94–1.00 vs. 0.97, 95% CI 0.93–1.00, *p* = 0.13). It was noteworthy that the studies with real-time RT-PCR as reference standard displayed a lower sensitivity (0.96, 95% CI 0.94–0.99 vs. 0.99, 95s% CI 0.97–1.00, *p* = 0.26) and a higher specificity than RT-PCR (0.98, 95% CI 0.96–1.00 vs. 0.96, 95% CI 0.90–1.00, *p* = 0.07). Compared to RT-RPA, RT-LAMP showed almost the same sensitivity (0.97, 95% CI 0.95–1.00 vs. 0.97, 95% CI 0.95–1.00, *p* = 0.19) and specificity (0.98, 95% CI 0.94–1.00 vs. 0.98, 95% CI 0.94–1.00, *p* = 0.42). Among the several covariates above, there was no significant factor affecting heterogeneity observed in the Joint model (*p* > 0.05) (Table 3 and Supplementary File Figure S2B).

Further influential analysis using a bivariate box plot and Cook’s distance revealed an outlier study for ICA-based POCTs and four outliers for NAIA-based POCTs (Supplementary File Figures S3 and S4). After removing these outliers [21,35,36,37,41], the pooled sensitivities of ICA- and NAIA-based POCTs for detecting PEDV showed significantly statistical differences comparing with those of the corresponding overall studies (0.94, 95% CI 0.92–0.97 vs. 0.95, 95% CI 0.93–0.97, *p* = 0.00 for ICA-based POCTs; 0.98, 95% CI 0.96–1.00 vs. 0.97, 95% CI 0.95–0.99 for NAIA-based POCTs, *p* = 0.01), indicating that these outliers may also have contributed to heterogeneity among the studies.

### 3.5. Clinical Utility

**Figure 6:**
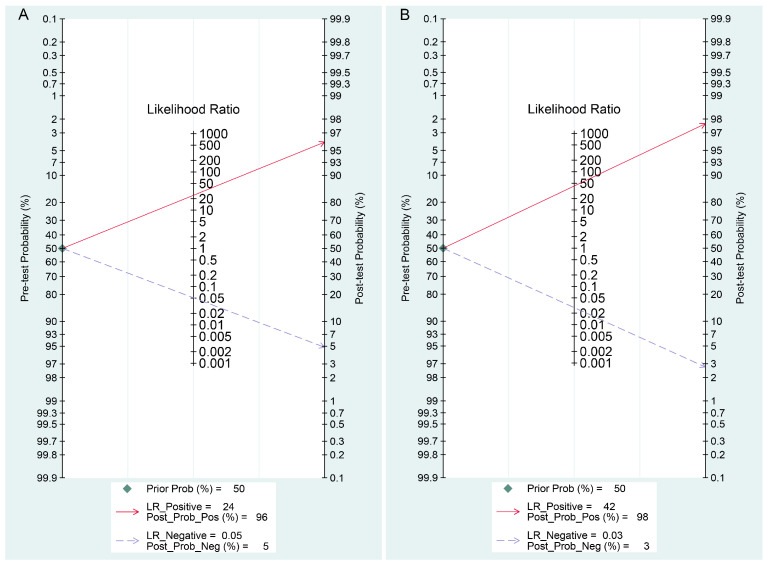
Fagan’s nomogram assessing the clinical diagnostic value of (**A**) ICA- and (**B**) NAIA-based POCTs for PEDV testing.

**Figure 7:**
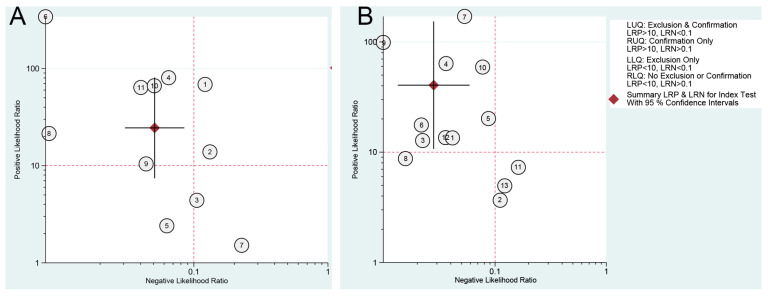
Likelihood ratio scattergrams of (**A**) ICA- and (**B**) NAIA-based POCTs for PEDV testing.

Fagan’s nomogram was used to assess the clinical diagnostic value of ICA- and NAAT-based POCTs for detecting PEDV. As shown in Figure 6, both ICA- and NAAT-based POCTs were demonstrated to have excellent diagnostic accuracy in clinical utility for the diagnosis of PEDV. At a pre-test probability of 50%, the post-test probability of positive results increased to 96% and negative results reduced to 5% for ICA-based POCTs (Figure 6A), respectively, suggesting that swine that are under suspicion of PEDV infections have 96% probability of having the disease when ICA-based POCTs indicate a positive result, and swine that are under suspicion of PEDV infection have a 5% probability of having the disease when the test result for ICA-based POCTs is negative. For NAAT-based POCTs, it was very informative, raising the probability of PEDV infection to 98% from 50% when positive and lowering the probability of disease to as low as 3% when negative, respectively (Figure 6B). In addition, the likelihood ratio scattergrams of both ICA- and NAIA-based POCTs showed summary point estimate of likelihood ratios obtained as functions of mean sensitivity and specificity in the left upper quadrant, suggesting that the two tests are useful for both the confirmation and exclusion of PEDV infection (Figure 7).

## 4. Discussion

ICA-based POCTs are low-cost, simple-to-use, and rapid assays without the need for specialized and costly equipment, which have been largely used in clinical diagnosis as a screening test for a variety of clinical markers. Traditionally, the ICA results are judged by a visual colorimetric measurement using the naked eye; the final result can depend upon the interpretation of the user. Thus, the problems for false positive and false negative are inevitable owing to this qualitative or semiquantitative decision [42]. The immunochromatography detection limit has recently been improved by using novel fluorescence materials for the preparation of test strips. One typical example is that an ICA based on a europium nanoparticle (EuNP)-monoclonal antibody fluorescent probe shows significantly higher sensitivity than that of a AuNP- based ICA [22]. In addition, some ICA-based tests provide qualitative information on the presence or absence of PEDV with the recent development of several instruments developed to measure the intensity of the test line for a more quantitative judgment through attaching to optic or electrochemical detectors. In this systematic review and meta-analysis, we identified 11 articles of ICA-based POCTs published since 2015, and 2 of them used portable strip readers to analyze the detection results of ICA [19,21]. Moreover, a smartphone camera and immunofluorescent analyzer were also applied to quantify the results of ICA for detecting PEDV [18,22]. These handheld instruments provide convenient tools for reading the information of the ICA strip and make the results more precise, objective, and sensitive.

For diagnostic meta-analysis, *I*^2^ value is considered to be an indicator of heterogeneity among studies, which quantifies the effect of heterogeneity and does not depend on the number of studies or the type of outcome data. *I*^2^ values of 25%, 50%, and 75% represent low, moderate, and high heterogeneity, respectively [14,43]. One of the primary causes of heterogeneity in test accuracy studies is threshold effect, which arises when differences in sensitivities and specificities or LRs occur due to different cut-offs or thresholds used in different studies to define a positive (or negative) result. A typical pattern of a “shoulder arm” plot in a SROC space or the Spearman’s correlation coefficient r ≥ 0.6 generally indicate a threshold effect [44,45]. In this meta-analysis for ICA-based POCTs, the high *I*^2^ values of both the sensitivity (*I*^2^ = 65.17%) and specificity (*I*^2^ = 92.78%) indicated significant heterogeneity, but the proportion of heterogeneity likely due to threshold effect was low (0.17) and the Spearman correlation coefficient was −0.209 (< 0.6), and no typical pattern of “shoulder arm” was observed in the SROC plot (Figure 4A), indicating that the heterogeneity may be caused by other factors except for threshold effect.

To further examine the potential sources of heterogeneity in the studies of ICA-based POCTs for PEDV, subgroup analysis and meta-regression were performed with the following covariates: (i) sample type, (ii) sample size, and (iii) reference standard. Our results showed that there was no significant heterogeneity factor in our subgroup analysis in relation to sample type. It is well known that the fecal–oral route is believed to be the primary mode of PEDV transmission; feces are considered a rapidly obtainable and readily available sample for PEDV detection [18,46]. When comparing the diagnostic performance of ICA-based POCTs for different sample types, however, we found that the overall sensitivity of the feces sample was lower than that of other types of samples including rectal swab, small intestine, colostrum, and serum (0.95 vs. 0.96; *p* = 0.00), which revealed that the diagnostic performance of feces specimen was not necessarily superior to other specimens in ICA-based POCTs for PEDV infection. Some explanations for such performance may be drawn from our current understanding of PEDV infection. As previously reported, feces collected at 42 dpi were PED- negative by PCR, but small intestines were PCR-positive for PEDV [47], indicating the effect of sample type and timing of testing on the test results.

In molecular biological diagnosis, a variety of PCR methods have been developed to detect PEDV, such as a nanoparticle-assisted PCR assay [48], duplex real-time RT-PCR [49], and multiplex TaqMan probe-based real-time PCR [50]. However, these PCR-based assays require well-equipped facilities, possibly expensive instruments, and trained personnel that typically are not suitable for on-the-spot detection in field situations or primitive laboratories in rural areas and developing countries. Recently, various isothermal amplification techniques that do not require the sophisticated thermos cycling involved in PCR have been developed as alternatives to PCR [51,52]. Alternate novel isothermal amplification techniques such as RT-LAMP and RT-RPA are rapid and do not require expensive equipment such as a thermal cycler, and hence can be performed in a point-of-care context or resource-poor laboratories. In our current meta-analysis, 7 of 13 studies with NAIA-based POCT used an RT-LAMP assay [29,30,33,35,37,40,41] and 6 studies adopted an RT-RPA assay to detect PEDV [31,32,34,36,38,39]. In general, the sensitivities of nucleic acid tests are in the order of real-time RT-PCR followed by RT-LAMP, RT-RPA, and conventional RT-PCR [53]. Our results demonstrated that RT-LAMP had equal high sensitivity to RT-RPA for the detection of PEDV and was comparable in specificity to RT-RPA; even the detection rate of PEDV by RT–LAMP was higher than that by real-time RT-PCR [29]. Compared to RT-LAMP, RT-RPA has more advantages in incubation temperature (about 37 °C) and incubation time (about 40 min), while RT-LAMP requires about 65 °C and 1 h in incubation temperature and time, respectively [53]. Additionally, RT-LAMP and RT-RPA techniques incorporating with devices such as strip [39,40] and microfluid chip [30,35] have made the assays more convenient and suitable for use in point-of-care testing for PEDV. Notably, CRISPR-based isothermal assay has shown great promise due to its high sensitivity, specificity, and reliability in the development of next-generation POC molecular diagnostic technologies for PEDV [32].

In this analysis, NAIA-based POCTs displayed better diagnostic performance when compared with ICA-based POCTs. Particularly, ICA-based POCTs are most likely to have low sensitivity in samples with low viral loads. Nevertheless, due to methodological limitations and the lack of direct head-to-head comparisons of the two test types, and other unknown factors, it is not possible to state with any certainty whether NAIA-based POCTs are superior to ICA-based POCTs. Moreover, ICA-based POCTs have the advantages of shorter test time, and are easy-to-use and cost-effective, especially in low- and middle-income countries because of their advantage of relative cheapness as compared with NAIA-based POCTs. Meanwhile, the benefits of administering ICA-based POCTs in suspected cases are the rapid diagnosis for clinical treatment and management and the ability to quarantine PEDV-infected individuals in time effectively, which has given the excellent prospects in PEDV epidemic monitoring and control.

The advantage of this study is that it is a first comprehensive meta-analysis of published studies on evaluating performance of ICA- and NAIA-based POCTs for detecting PEDV. However, several significant limitations should be considered. First, most of the diagnostic studies enrolled samples from field farms with diarrhea outbreaks and were not blind-designed, which might lead to an overestimation of the diagnostic value. Among 24 studies for inclusion in this review, 6 studies used the samples that confirmed PEDV definitely prior [18,22,30,33,36,37] and only one study provided relatively detailed clinical information of fecal samples [34]. Second, the number of studies enrolled in this analysis is still relatively small, which might limit the power of regression to detect significant effects and lead to biased results. Third, the significant publication bias was found in the studies for NAIA-based POCTs, suggesting that studies with positive results are more likely to be published than studies illustrating more negative results, which may be one possible reason of variations among studies and may limit the strength of conclusions that we are currently able to draw; thus, the results we report should be interpreted with a high degree of caution. Future real-world high-quality research iss needed to further assess the performance of ICA- and NAIA-based POCTs in clinical application and compare evidence across large-scale studies.

## 5. Conclusions

The current systematic review and meta-analysis is the first to establish an overview of the diagnostic accuracy of ICA- and NAIA-based POCTs for detecting PEDV. Our results revealed that NAIA-based POCTs had a better diagnostic performance than ICA-based POCTs, and both of the two tests had satisfactory diagnostic accuracy for almost all the technologies evaluated for diagnostic performance and accuracy. However, it is vital to acknowledge that the current data may be not generalizable due to a number of factors. In future, more high-quality studies, following rigorous methodologies that address rapid POCTs for PEDV, will be needed in order to evaluate the utility of these tests in veterinary clinical practice, as well as strengthening the confidence in relation to the accuracy of the tests.

## Figures and Tables

**Table 1:** Characteristics of the studies included in the meta-analysis.

Study (First Author, Year)	Country	Sample Type	Sample Size	Reference Standard	TP	FP	FN	TN
ICA-based POCTs								
Zou, 2021 [18]	China	feces	108	RT-PCR	62	0	8	38
Wang, 2021 [19]	China	rectal swab	160	real-time RT-PCR	71	5	10	74
Liu, 2020a [20]	China	colostrum	63	ELISA	51	4	2	6
Liu, 2020b [21]	China	rectal swab	923	RT-PCR	63	2	0	858
Xu, 2020 [22]	China	feces	60	RT-PCR	33	5	3	19
Zhang, 2020 [23]	China	feces	80	RT-PCR	36	0	2	42
Bian, 2019 [24]	China	feces	27	RT-PCR	20	3	2	2
Jia, 2019 [25]	China	feces	115	RT-PCR	62	2	0	51
Li, 2018 [26]	China	serum	201	ELISA	120	7	5	69
Lyoo, 2017 [27]	Korea	rectal swab	130	real-time RT-PCR	57	1	3	69
Kim, 2015 [28]	Korea	feces	493	real-time RT-PCR	218	4	9	262
NAIA-based POCTs								
Kim, 2021 [29]	Korea	feces and intestine	149	real-time RT-PCR	95	4	2	48
El-Tholoth, 2021 [30]	Egypt	rectal swabs	11	real-time RT-PCR	5	0	0	6
Li, 2021 [31]	China	feces and intestine	45	real-time RT-PCR	13	0	0	32
Yang, 2021 [32]	China	feces and intestine	15	real-time RT-PCR	5	0	0	10
Di, 2021 [33]	China	feces and lymph nodes	60	RT-PCR	12	3	0	45
Wang, 2020 [34]	China	not stated	80	real-time RT-PCR	71	0	1	8
Zhou, 2020 [35]	China	feces and intestine	173	real-time RT-PCR	80	0	4	89
Wang, 2019 [36]	China	not stated	65	RT-PCR	35	3	0	27
Mai, 2018 [37]	Japan	feces	99	RT-PCR	50	0	0	49
Wang, 2018 [38]	China	intestine	76	real-time RT-PCR	42	0	3	31
Wang, 2016 [39]	China	feces	41	RT-PCR	6	4	1	30
Gou, 2015 [40]	China	not stated	20	RT-PCR	14	0	0	6
Yu, 2015 [41]	China	feces and intestine	52	real-time RT-PCR	27	4	3	18

Note: TP = true positive; FP = false positive; FN = false negative; TN = true negative.

**Table 2:** Summary estimates of the diagnostic accuracy of ICA- and NAIA-based POCTs used to detect PEDV.

	Sensitivity (95% CI)	Specificity (95% CI)	PLR (95% CI)	NLR (95% CI)	DOR (95% CI)	AUC (95% CI)
ICA-based POCTs	0.95	0.96	24.5	0.05	480	0.98
	(0.92–0.97)	(0.88–0.99)	(7.4–81.0)	(0.03–0.08)	(111–2074)	(0.96–0.99)
NAIA-based POCTs	0.97	0.98	42.4	0.03	1517	0.99
	(0.94–0.99)	(0.91–0.99)	(10.9–164.9)	(0.01–0.06)	(290–7943)	(0.98–1.00)

Note: CI, confidence interval; PLR, positive likelihood ratio, NLR, negative likelihood ratio, DOR, diagnostic odds ratio.

**Table 3:** Subgroup analysis and meta-regression for the pooled sensitivity and specificity of ICA-and NAIA-based POCTs according to study design.

Parameter	Category	No. of Studies	Sensitivity		Specificity		LRT Chi-Square	*P* (Joint Model)
			Pooled Value (95% CI)	*p*1	Pooled Value (95% CI)	*p*2		
ICA-based POCTs								
Sample type	Feces	6	0.95 (0.91–0.98)	0.00	0.96 (0.90–1.00)	0.56	0.21	0.90
	Other ^a^	5	0.96 (0.92–0.99)		0.96 (0.90–1.00)			
Sample size	≥100	7	0.96 (0.93–0.98)	0.05	0.98 (0.96–1.00)	0.02	5.12	0.08
	<100	4	0.94 (0.89–0.99)		0.82 (0.60–1.00)			
Reference standard	ELISA	2	0.96 (0.92–1.00)	0.19	0.81 (0.46–1.00)	0.21	3.95	0.14
	Other ^b^	9	0.94 (0.92–0.97)		0.97 (0.94–1.00)			
NAIA-based POCTs								
Sample type	Feces	8	0.97 (0.94–0.99)	0.10	0.97 (0.93–1.00)	0.44	0.59	0.74
	Other ^c^	5	0.98 (0.95–1.00)		0.98 (0.94–1.00)			
Sample size	≥100	2	0.97 (0.93–1.00)	0.18	0.98 (0.94–1.00)	0.13	0.18	0.91
	<100	11	0.98 (0.95–1.00)		0.97 (0.93–1.00)			
Reference standard	Real-time RT-PCR	8	0.96 (0.94–0.99)	0.26	0.98 (0.96–1.00)	0.07	3.85	0.15
	RT-PCR	5	0.99 (0.97–1.00)		0.96 (0.90–1.00)			
Assaytype	RT-LAMP	7	0.97 (0.95–1.00)	0.19	0.98 (0.94–1.00)	0.42	0.02	0.99
	RT-RPA	6	0.97 (0.95–1.00)		0.98 (0.94–1.00)			

Note: CI, confidence interval; ^a^, rectal swab, small intestine, colostrum, or serum; ^b^, RT-PCR and real-time RT-PCR; ^c^, rectal swab, intestine, or not stated.

## Data Availability

All of the detailed information regarding the quality assessment, regression analysis and program commands used in Stata software are available in the Supplementary Materials.

## Supplementary Materials

The following supporting information can be downloaded at: https://www.mdpi.com/article/10.3390/v14071355/s1, Figure S1: HSROC plots for (A) ICA- and (B) NAIA-based POCTs in detecting PEDV; Figure S2: Forest plot of multiple univariable stratified meta-regression and subgroup analyses of (A) ICA- and (B) NAIA-based POCTs for PEDV testing; Figure S3: Bivariate box plot for ICA-based POCTs (A) and NAIA-based POCTs (B) for detecting PEDV. The bivariate box plot describes the degree of interdependence including the central location and identification of any outliers. The inner oval represents the median distribution of the data points. The outer oval represents the 95% confidence bound; Figure S4: Influential analysis for the studies of (A) ICA- and (B) NAIA-based POCTs for PEDV testing; Table S1: Quality Assessment of Diagnostic Accuracy Studies 2 (QUADAS-2) summary items for risk of bias (A) and applicability concerns (B) for all studies. References [18,20,22,26,29,30,33,36,37,38,39,40] are cited in the supplementary materials.
