# Supplementary material for: Point-of-Care Tests for Rapid Detection of Porcine Epidemic Diarrhea Virus: A Systematic Review and Meta-Analysis
Source: Viruses. 2022 Jun 21;14(7):1355. doi: 10.3390/v14071355 (PMC9321219; doi:10.3390/v14071355)
Supplement: Supplementary file 1 [file viruses-14-01355-s001.zip › viruses-1745883-supplementary.pdf]

Supplementary Material

Quality assessment

Results of QUADAS-2 assessment for evaluating the quality of the studies are shown in Table S1. For ICA-based POCTs, sample selection procedures were mostly at low risk of bias, considering that most studies (10/11, 90.9%) specified that the samples were obtained from field farms. Only one study had high risk of sample selection bias as the samples had been identified in laboratory [1]. In the index test domain, 81.8% studies (9/11) were classified as low risk of bias since the authors indicated that their index and reference tests were done simultaneously/in parallel to each other and the index test results were interpreted without knowledge of the results of the reference standard, the remaining two studies were classified as high risk of bias [1, 2]. Regarding the reference standard, two studies were judged to be at high risk of bias as they used ELISA as reference standard [3, 4], and the rest of included studies were designated as having low risk of bias. In the flow and timing domain, most studies have a low risk of flow and timing bias with the exception that two studies were at high risk of bias as the samples for the reference test and the index test were not taken at the same time [1, 2].

For NAIA-based POCTs, regarding the patient selection domain, four studies were scored as having high risk of bias because they reported that the samples used for evaluation were obtained from laboratory but not clinical sampling [5-8], also they were classified as high risk of bias in the index test domain and flow and timing domain since the index test results were identified with knowledge of the results of the reference standard. Two studies provided no information on whether the samples for the reference standard and the index test were tested simultaneously and were marked as having unknown risk of index test bias. [9, 10]. In the reference standard domain, we graded 8 studies as having low risk of bias as they used real-time RT-PCR as reference standard, 5 studies that used (non-quantitative) RT-PCR were marked as having high risk of bias [6-8, 11, 12].

With regard to applicability, the sample selection domain was assessed to be high concern for five of the studies (1 for ICA-based POCTs [1], 4 for NAIA-based POCTs [5-8]) as they enrolled known samples from laboratory but not field. Regarding the reference standard domain, 2 studies were assessed to be high concern of standard test applicability due to the usage of ELISA as reference test for ICA-based POCTs [3, 4], and 5 studies that used (non-quantitative) RT-PCR were graded as having high concern of standard test applicability for NAIA-based POCTs [6-8, 11, 12]. No studies are considered to be at risk of bias in the index test domain. Table S1 presents the risk of bias assessment (A) and applicability concerns (B) of individual studies in the meta-analysis.

Table S1. Quality Assessment of Diagnostic Accuracy Studies 2 (QUADAS-2) summary items for risk of bias (A) and applicability concerns (B) for all studies.

A

Study	Sample Selection	Index Test: NAIA-Based	Index Test: ICA-Based POCTs	Reference Standard	Flow and Timing
**ICA-Based POCTs**					
Zou et al. 2021	Low		High	Low	High
Wang et al. 2021	Low		Low	Low	Low
Liu et al. 2020a	Low		Low	High	Low
Liu et al. 2020b	Low		Low	Low	Low
Xu et al. 2020	High		High	Low	High
Zhang et al. 2020	Low		Low	Low	Low
Bian et al. 2019	Low		Low	Low	Low
Jia et al. 2019	Low		Low	Low	Low
Li et al. 2018	Low		Low	High	Low
Lyoo et al. 2017	Low		Low	Low	Low
Kim et al. 2015	Low		Low	Low	Low
**NAIA-Bases POCTs**					
Kim et al. 2021	Low	Unclear		Low	Low
El-Tholoth et al. 2021	High	High		Low	High
Li et al. 2021	Low	Low		Low	Low
Yang et al. 2021	Low	Low		Low	Low
Di et al. 2021	High	High		High	High
Wang et al. 2020	Low	Low		Low	Low
Zhou et al. 2020	Low	Low		Low	Low
Wang et al. 2019	High	High		High	High
Mai et al. 2018	High	High		High	High
Wang et al. 2018	Low	Unclear		Low	Low
Wang et al. 2016	Low	Low		High	Low
Gou et al. 2015	Low	Low		High	Low
Yu et al. 2015	Low	Low		Low	Low

B

Study	Sample Selection	Index Test: NAIA-Based Tests	Index Test: ICA-Based Tests	Reference Standard
**ICA-Based Tests**				
Zou et al. 2021	Low		Low	Low
Wang et al. 2021	Low		Low	Low
Liu et al. 2020a	Low		Low	High
Liu et al. 2020b	Low		Low	Low
Xu et al. 2020	High		Low	Low
Zhang et al. 2020	Low		Low	Low
Bian et al. 2019	Low		Low	Low
Jia et al. 2019	Low		Low	Low
Li et al. 2018	Low		Low	High
Lyoo et al. 2017	Low		Low	Low
Kim et al. 2015	Low		Low	Low
**NAIA-Bases Tests**				
Kim et al. 2021	Low	Low		Low
El-Tholoth et al. 2021	High	Low		Low
Li et al. 2021	Low	Low		Low
Yang et al. 2021	Low	Low		Low
Di et al. 2021	High	Low		High
Wang et al. 2020	Low	Low		Low
Zhou et al. 2020	Low	Low		Low
Wang et al. 2019	High	Low		High
Mai et al. 2018	High	Low		High
Wang et al. 2018	Low	Low		Low
Wang et al. 2016	Low	Low		High
Gou et al. 2015	Low	Low		High
Yu et al. 2015	Low	Low		Low

Figure S1. HSROC plots for (A) ICA- and (B) NAIA-based POCTs in detecting PEDV.

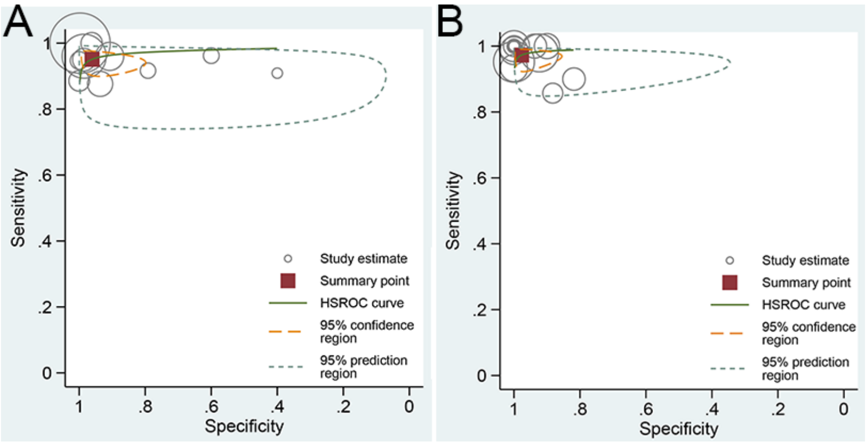

Figure S2. Forest plot of multiple univariable stratified meta-regression and subgroup analyses of (A) ICA- and (B) NAIA-based POCTs for PEDV testing.

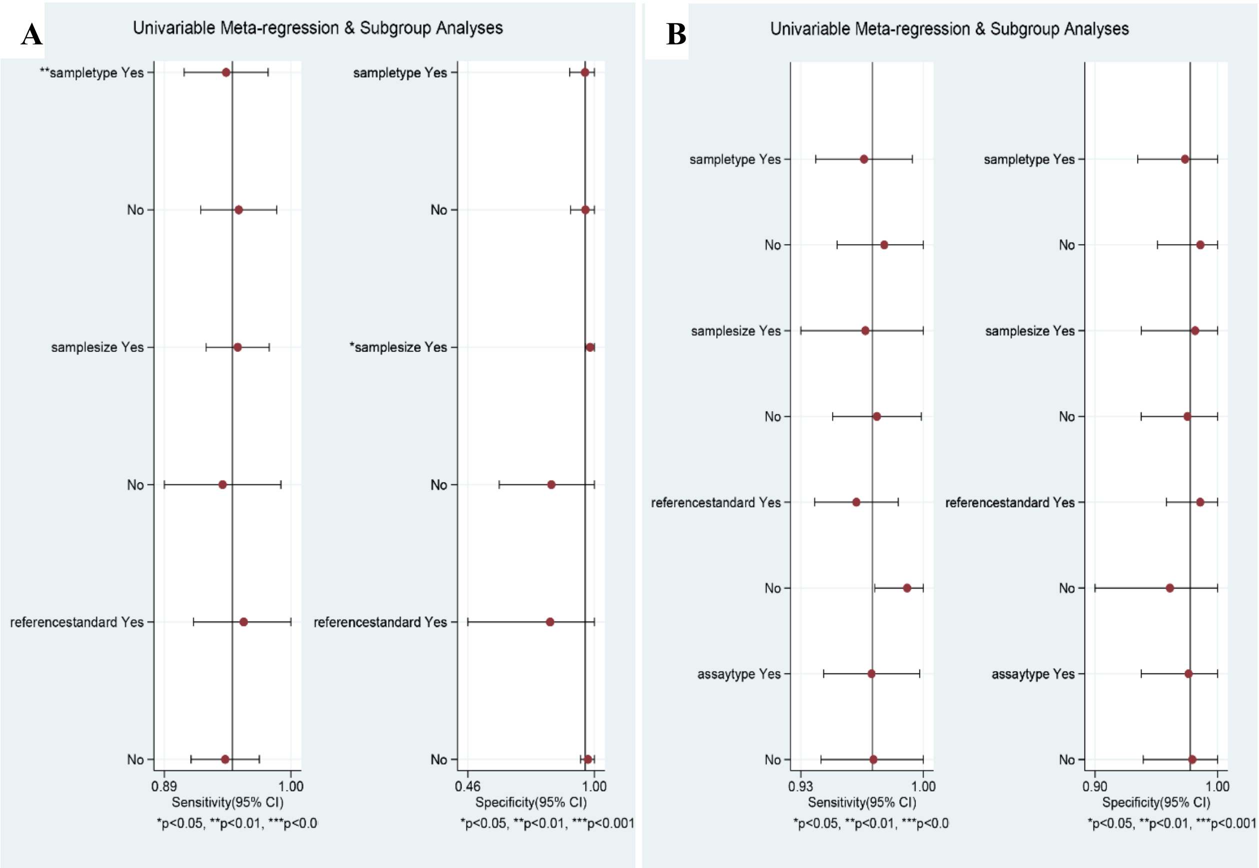

Figure S3. Bivariate box plot for ICA-based POCTs (A) and NAIA-based POCTs (B) for detecting PEDV. The bivariate box plot describes the degree of interdependence including the central location and identification of any outliers. The inner oval represents the median distribution of the data points. The outer oval represents the 95% confidence bound.

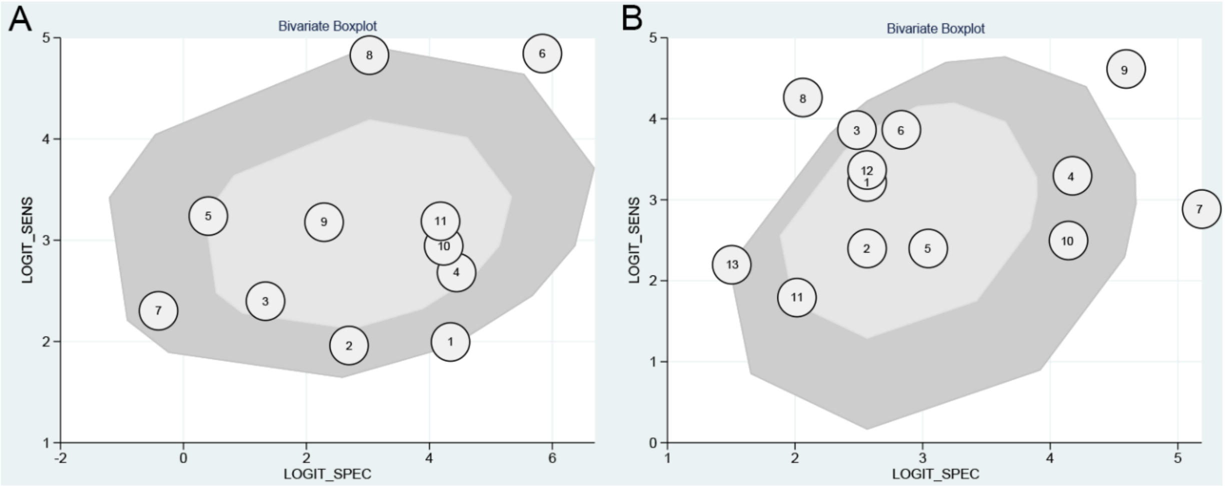

Figure S4. Influential analysis for the studies of (A) ICA- and (B) NAIA-based POCTs for PEDV testing. **a** goodness of fit; **b** Bivariate normality; **c** Influence analysis; **d** Outlier detection.

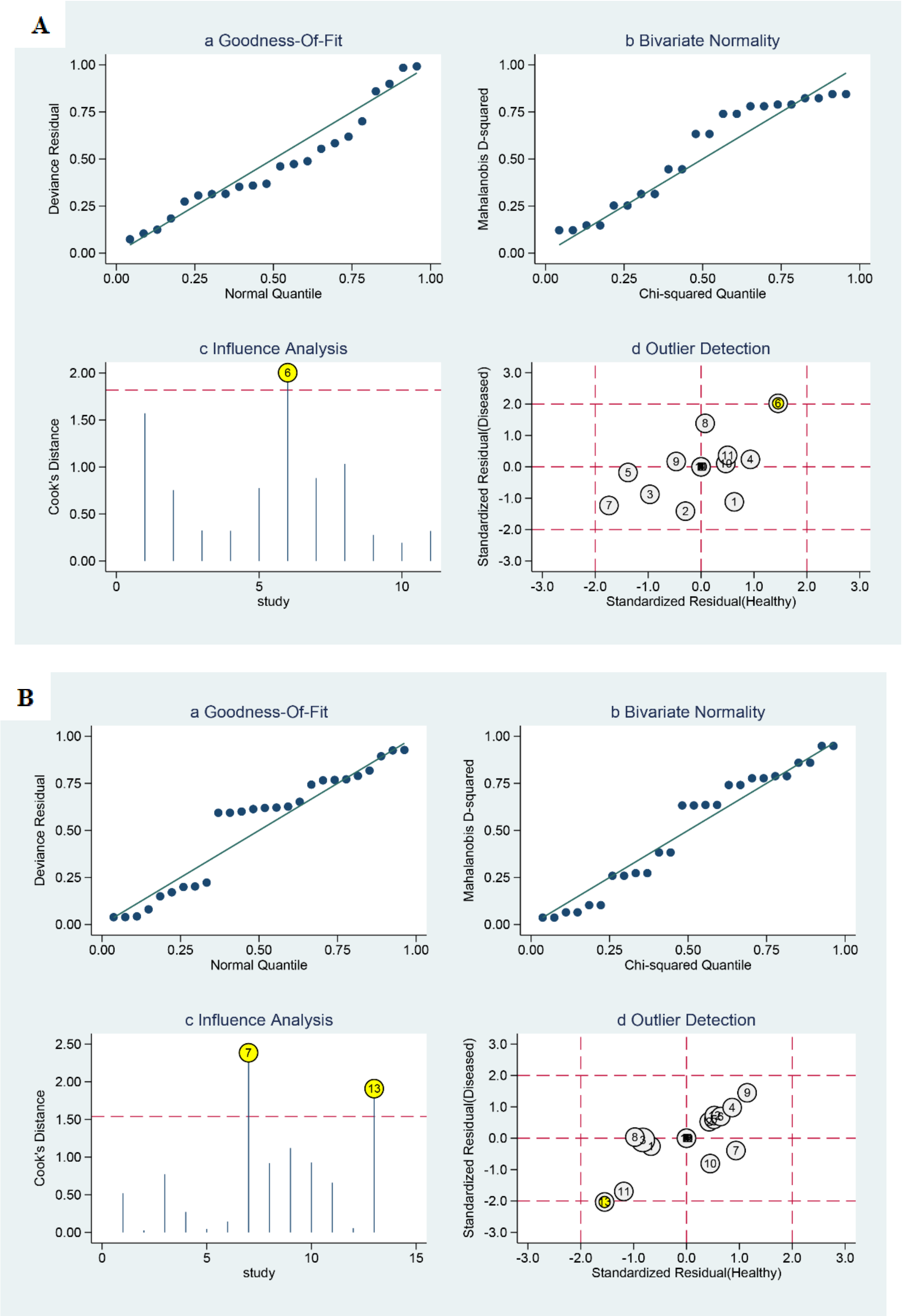

The whole process of data analysis and commands in Stata was as follows:

```
# install the packages for diagnostic meta-analysis in Stata 17.0
ssc install midas
ssc install metandi
# details of the data set
describe
# get the values for sensitivity, specificity, positive likelihood ratio, negative likelihood ratio, diagnostic odds ratio, and threshold effect
midas tp fp fn tn, res(all)
# get summary ROC curve and the area under the curve
midas tp fp fn tn, plot sroc(both)
# hierarchical summary receiver operating characteristic curve
metandi tp fp fn tn, plot
# bivariate box plot describes the degree of interdependence including the central location and identification of any outliers
midas tp fp fn tn, bivbox
# heterogeneity statistics
midas tp fp fn tn, es(x) table(dss)
# univariable meta-regression & subgroup analyses
midas tp fp fn tn, reg(sampletype samplesize referencestand assaytype)
# linear regression test of funnel plot asymmetry for investigating publication bias
midas tp fp fn tn, pubbias
# fagan plot (Bayes nomogram)
midas tp fp fn tn, fagan(0.5)
# likelihood ratio scattergram
midas tp fp fn tn, lrmat
```

References

1. Xu, F.; Jin, Z.; Zou, S.; Chen, C.; Song, Q.; Deng, S.; Xiao, W.; Zhang, X.; Jia, A.; Tang, Y. EuNPs-mAb fluorescent probe based immunochromatographic strip for rapid and sensitive detection of porcine epidemic diarrhea virus. *Talanta.* **2020,** 214, 120865.
2. Zou, S.; Wu, L.; Li, G.; Wang, J.; Cao, D.; Xu, T.; Jia, A.; Tang, Y. Development of an accurate lateral flow immunoassay for PEDV detection in swine fecal samples with a filter pad design. *Anim. Dis.* **2021,** 1, 27.
3. Liu, J.; Gao, R.; Shi, H.; Cong, G.; Chen, J.; Zhang, X.; Da, S.; Cao, L.; Wang, X.; Zhang, J., et al. Development of a rapid immunochromatographic strip test for the detection of porcine epidemic diarrhea virus specific SIgA in colostrum. *J. Virol. Methods.* **2020,** 279, 113855.
4. Li, R.; Tian, X.; Yu, Y.; Qiao, S.; Wang, Z.; Ma, J. Development of a rapid immunochromatographic assay for detection of antibodies against porcine epidemic diarrhea virus. *Pol. J. Vet. Sci.* **2018,** 21, 139-147.
5. El-Tholoth, M.; Bai, H.; Mauk, M. G.; Saif, L.; Bau, H. H. A portable, 3D printed, microfluidic device for multiplexed, real time, molecular detection of the porcine epidemic diarrhea virus, transmissible gastroenteritis virus, and porcine deltacoronavirus at the point of need. *Lab. Chip.* **2021,** 21, 1118-1130.

6. Di, Y.; Tan, F.; Qin, S.; Yu, X.; Cui, W.; Jiang, Y.; Wang, L.; Tang, L.; Zhou, H.; Wang, X., et al. Establishment and application of RT-LAMP rapid detection assay for porcine epidemic diarrhea virus. *Chin. J. Prev. Vet. Med.* **2021,** 43, 832-837.
7. Wang, X.; Xu, X.; Hu, W.; Zuo, K.; Li, Z.; Kan, Y.; Yao, L.; Ji, J.; Bi, Y. Visual detection of porcine epidemic diarrhea virus using a novel reverse transcription polymerase spiral reaction method. *BMC Vet. Res.* **2019,** 15, 116.
8. Mai, T. N.; van Nguyen, D.; Yamazaki, W.; Okabayashi, T.; Mitoma, S.; Notsu, K.; Sakai, Y.; Yamaguchi, R.; Norimine, J.; Sekiguchi, S. Development of pooled testing system for porcine epidemic diarrhoea using real-time fluorescent reverse-transcription loop-mediated isothermal amplification assay. *BMC Vet. Res.* **2018,** 14, 172.
9. Kim, J.-K.; Kim, H.-R.; Kim, D.-Y.; Kim, J.-M.; Kwon, N.-Y.; Park, J.-H.; Park, J.-Y.; Kim, S.-H.; Lee, K.-K.; Lee, C., et al. A simple colorimetric detection of porcine epidemic diarrhea virus by reverse transcription loop-mediated isothermal amplification assay using hydroxynaphthol blue metal indicator. *J. Virol. Methods.* **2021,** 298, 114289.
10. Wang, J.; Zhang, R.; Wang, J.; Han, Q.; Liu, L.; Li, Y.; Yuan, W. Real-time reverse transcription recombinase polymerase amplification assay for rapid detection of porcine epidemic diarrhea virus. *J. Virol. Methods.* **2018,** 253, 49-52.
11. Wang, F.-X.; Yuan, D.-Y.; Jin, Y.-N.; Hu, L.; Sun, Z.-Y.; He, Q.; Zhao, S.-H.; Zhan, S.-B.; Wen, Y.-J. Reverse transcription cross-priming amplification-nucleic acid test strip for papid detection of porcine epidemic diarrhea virus. *Sci. Rep.* **2016,** 6, 24702.
12. Gou, H.; Deng, J.; Wang, J.; Pei, J.; Liu, W.; Zhao, M.; Chen, J. Rapid and sensitive detection of porcine epidemic diarrhea virus by reverse transcription loop-mediated isothermal amplification combined with a vertical flow visualization strip. *Mol. Cell. Probes.* **2015,** 29, 48-53.
